# Temocillin for the Treatment of Urinary Tract Infections: First Italian Data from a Multicentre Real-World Experience

**DOI:** 10.3390/antibiotics15101034

**Published:** 2026-10-17

**Authors:** Nicola Benvenuto, Massimiliano Lanzafame, Sergio Venturini, Verena Zerbato, Arianna Perosa, Giovanni Mori, Giovanni Del Fabro, Tommaso Cai, Chiara Zanusso, Filippo Giorgio Di Girolamo, Stefano Colla, Valentina Gerussi, Vasiliki Koumaki, Marina Busetti, Stefano Di Bella

**Affiliations:** 1 Infectious Diseases Unit, University Hospital of Trieste (ASUGI), 34125 Trieste, Italy; nicola.benvenuto98@gmail.com (N.B.); verena.zerbato@gmail.com (V.Z.); valentina.gerussi@asugi.sanita.fvg.it (V.G.); 2 Infectious Diseases Unit, Santa Chiara Hospital, Azienda Sanitaria Universitaria Integrata del Trentino (ASUIT), 38122 Trento, Italy; massimiliano.lanzafame@apss.tn.it (M.L.);; 3 Centre for Medical Sciences (CISMed), University of Trento, 38122 Trento, Italy; tommaso.cai@apss.tn.it; 4 Infectious Diseases Unit, Azienda Sanitaria Friuli Occidentale (ASFO), 33170 Pordenone, Italy; sergio.venturini@asfo.sanita.fvg.it (S.V.); giovanni.delfabro@asfo.sanita.fvg.it (G.D.F.); 5 School of Medicine, University of Trieste, 34149 Trieste, Italy; 6 Urology Unit, Santa Chiara Hospital, Azienda Sanitaria Universitaria Integrata del Trentino (ASUIT), 38122 Trento, Italy; 7 Pharmacy Unit, Azienda Sanitaria Friuli Occidentale (ASFO), 33170 Pordenone, Italy; chiara.zanusso@asfo.sanita.fvg.it; 8 Pharmacy Unit, University Hospital of Trieste (ASUGI), 34125 Trieste, Italy; filippogiorgio.digirolamo@asugi.sanita.fvg.it (F.G.D.G.); stefano.colla@asugi.sanita.fvg.it (S.C.); 9 Department of Microbiology, Medical School, National and Kapodistrian University of Athens, 11527 Athens, Greece; vkoumaki@gmail.com; 10 Microbiology Unit, University Hospital of Trieste (ASUGI), 34149 Trieste, Italy; marina.busetti@asugi.sanita.fvg.it; 11 Clinical Department of Medical, Surgical and Health Sciences, University of Trieste, 34149 Trieste, Italy

**Keywords:** temocillin, UTIs, MDROs, carbapenem-sparing, sepsis

## Abstract

**Background**: Temocillin is a narrow-spectrum β-lactam with activity against extended-spectrum β-lactamase (ESBL)- and AmpC-producing Enterobacterales and represents a potential carbapenem-sparing option, especially for urinary tract infections (UTIs). This multicentre study evaluated the real-world use, clinical outcomes, and tolerability of temocillin in Italian clinical practice. **Methods**: We retrospectively included adults treated with temocillin for UTIs, with or without concomitant bloodstream infection, for at least 5 days at three Italian hospitals between November 2024 and May 2026. Data were collected from electronic medical records. **Results**: The study included 30 patients. Median age was 51 years (IQR 39–74); 50% presented with sepsis and 40% had UTI-associated bacteraemia. Pyelonephritis was the most common presentation (53.3%). At least one positive culture was obtained in 76.7% of patients, yielding 28 isolates. Of these, 27 were Enterobacterales, and 21/27 (77.8%) were ESBL producers. *E. coli* accounted for 64.3% of all isolates. Temocillin was administered at 6 g/day in 80% of patients and by continuous infusion in 76.7%. Outpatient parenteral antimicrobial therapy (OPAT) using elastomeric pumps was used in 16.7% of patients. Clinical success at the end of the overall antimicrobial treatment course was achieved in 28/30 patients (93.3%; 95% CI 77.9–99.2%). No recurrence occurred within 30 days, and no treatment-related adverse events directly attributable to temocillin were recorded. Two patients developed *Clostridioides difficile* infection within 60 days after treatment completion, with no established causal relationship with temocillin. **Conclusions:** This first Italian multicentre real-world experience reports favourable clinical outcomes with temocillin in UTIs, including in selected patients with sepsis or UTI-associated bloodstream infection, and describes the use of continuous infusion and OPAT in appropriately selected patients.

## 1. Introduction

Antimicrobial resistance among Gram-negative bacteria has emerged as a major global public health concern, complicating the management of bacterial infections. In particular, multidrug-resistant Enterobacterales, including extended-spectrum β-lactamase (ESBL), AmpC, and carbapenemase-producing isolates, have become increasingly prevalent in both community and healthcare settings, posing significant therapeutic challenges [1]. The spread of these organisms has progressively reduced the effectiveness of conventional antimicrobial therapies, often requiring the use of carbapenems or novel β-lactam/β-lactamase inhibitor combinations [2,3]. While these agents have expanded the available therapeutic arsenal, their widespread use raises concerns regarding the further emergence and selection of resistant pathogens. Consequently, antimicrobial stewardship programs have increasingly focused on optimizing antibiotic use and promoting carbapenem-sparing strategies whenever clinically appropriate. In this scenario, renewed attention has been directed toward older antimicrobial agents with retained activity against multidrug-resistant Enterobacterales, such as temocillin, which may represent a valuable treatment option in selected clinical settings [4,5].

Temocillin is a semisynthetic 6-α-methoxy derivative of ticarcillin with a relatively narrow spectrum of activity, mainly directed against Enterobacterales [6,7]. Its stability against many clinically relevant β-lactamases, including ESBLs and AmpC enzymes, together with its limited activity against Gram-positive organisms, anaerobes and most non-fermenting Gram-negative bacilli, makes temocillin an attractive targeted option from an antimicrobial stewardship perspective [7]. In addition, temocillin is predominantly excreted unchanged in the urine, achieving high urinary concentrations and adequate penetration into prostatic tissue. These pharmacokinetic properties make it particularly suitable for the treatment of urinary tract infections (UTIs), including prostatitis [8,9,10]. Its favourable safety profile, together with the possibility of administration through outpatient parenteral antimicrobial therapy (OPAT), further supports its clinical reappraisal [6].

Against this background, the present multicentre study aimed to characterize the clinical and microbiological features of patients treated with temocillin for UTIs in Italy, and to describe clinical outcomes and tolerability, as well as its potential role as a carbapenem-sparing option within antimicrobial stewardship strategies.

## 2. Results

### 2.1. Baseline Characteristics (Table 1)

**Figure 1:**
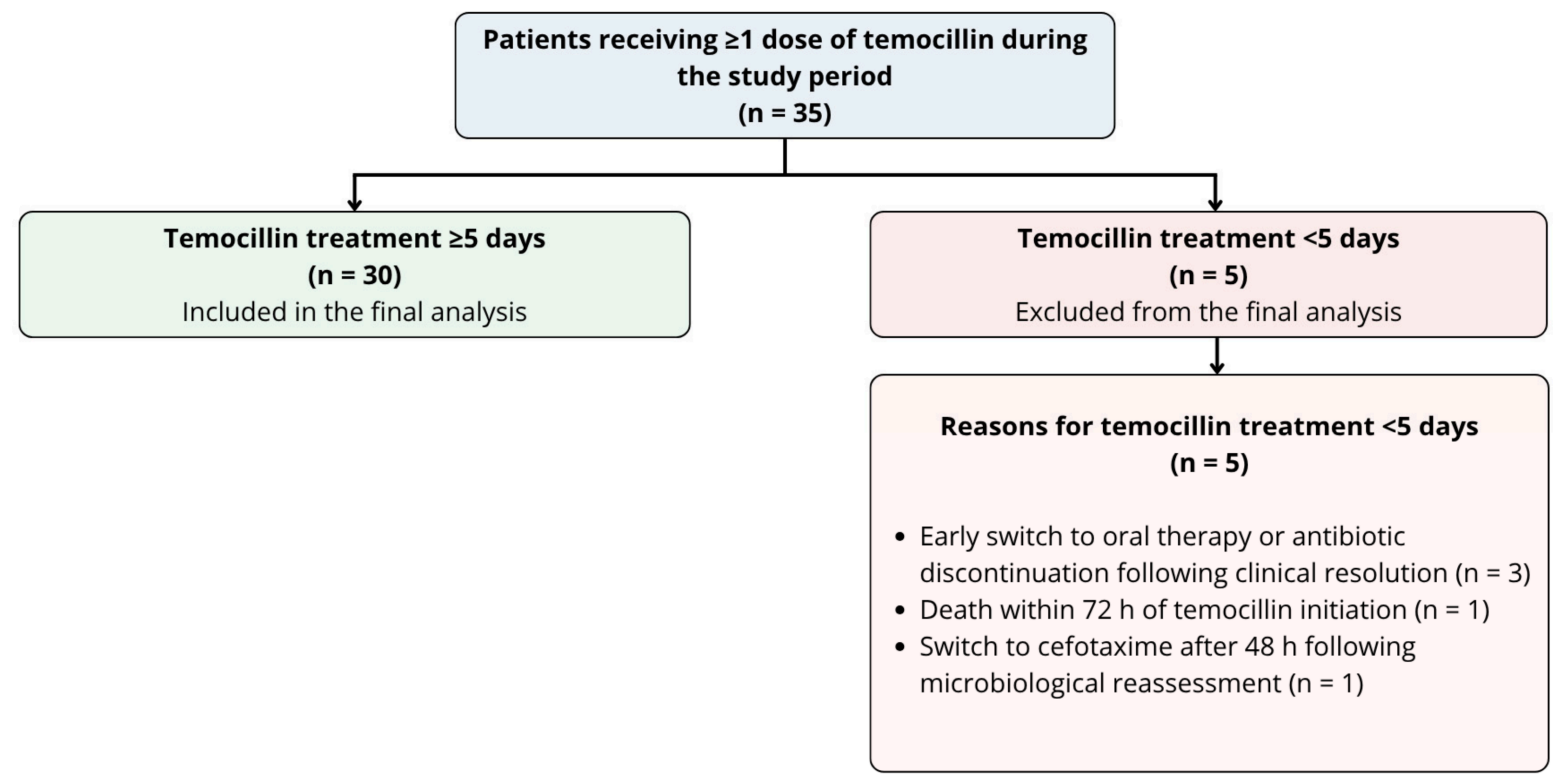
Patient selection flowchart. Flowchart of patient selection during the study period. Thirty-five patients received at least one dose of temocillin; 30 received temocillin for ≥5 days and were included in the final analysis, while 5 received temocillin for <5 days and were excluded. Reasons for treatment duration < 5 days are detailed in the figure.

During the study period, 35 patients received at least one dose of temocillin. Five patients received temocillin for <5 days and were excluded according to the predefined eligibility criteria (Figure 1), leaving 30 patients included in the final analysis. Of these, 15 patients (50%) were enrolled at the University Hospital (ASUGI) of Trieste, 7 (23.3%) at the Santa Maria degli Angeli Hospital of Pordenone, and 8 (26.7%) at the Santa Chiara Hospital of Trento.

**Table 1:** Baseline demographic, clinical, and laboratory characteristics of the study population.

Variable	Overall Cohort (*n* = 30)
Demographics	
Age (years)	51 (39–74)
Female (*n*, %)	18 (60)
CCI	2 (0–5)
Laboratory findings	
WBC (cells/mm^3^)	13,660 (10,658–17,558)
CRP (mg/L)	185 (100.5–227.5)
PCT (μg/L)	11.16 (0.80–26.55)
Creatinine (mg/dL)	0.80 (0.70–1.46)
Clinical presentation	
Pyelonephritis (*n*, %)	16 (53.3)
Febrile UTI (*n*, %)	10 (33.3)
Prostatitis (*n*, %)	3 (10)
Cystitis (*n*, %)	1 (3.3)
Sepsis (*n*, %)	15 (50)

Data are presented as median (interquartile range [IQR]) for continuous variables and as number (percentage) for categorical variables. Abbreviations: CCI, Charlson Comorbidity Index; CRP, C-reactive protein; PCT, procalcitonin; UTI, urinary tract infection; WBC, white blood cell count.

Overall, 18 patients (60%) were female and 12 (40%) were male. The median age was 51 years (IQR 39–74), while the median Charlson Comorbidity Index (CCI) was 2 (IQR 0–5). The median length of hospital stay was 10 days (IQR 9–14). At baseline, the median white blood cell count was 13,660 cells/mm^3^ (IQR 10,658–17,558), the median C-reactive protein (CRP) concentration was 185 mg/L (IQR 100.5–227.5), and the median procalcitonin (PCT) concentration was 11.16 μg/L (IQR 0.80–26.55). The median serum creatinine concentration was 0.80 mg/dL (IQR 0.70–1.46). A contemporaneous clinical diagnosis of sepsis was documented in 15 patients (50%); systematic retrospective confirmation according to Sepsis-3 SOFA criteria was not possible because complete SOFA data were unavailable. No patients were clinically diagnosed with septic shock during the index episode.

**Figure 2:**
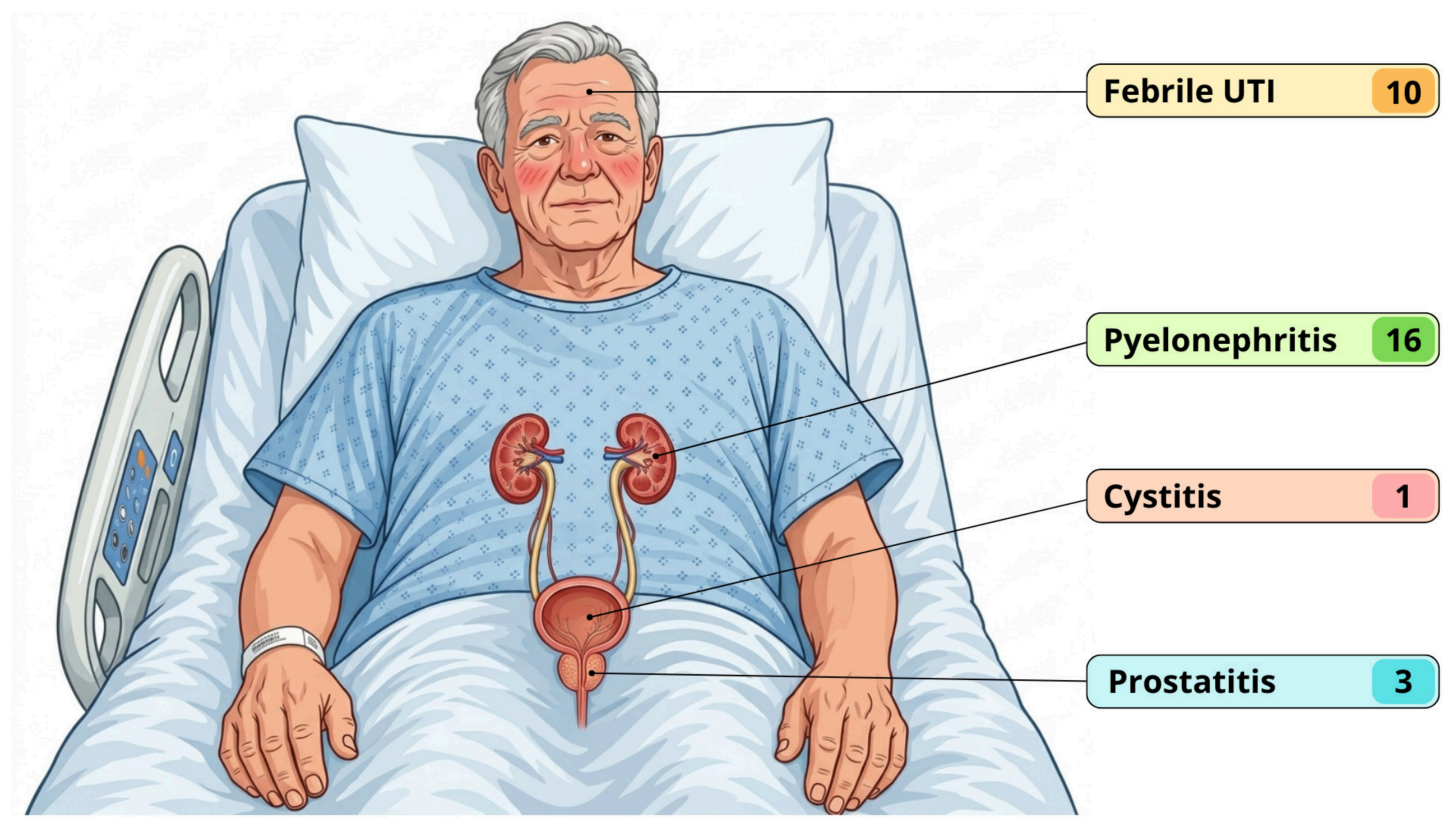
Distribution of urinary tract infection types in the study population. Numbers indicate the number of patients in each diagnostic category. Abbreviation: UTI, urinary tract infection.

According to the clinical presentation, 16 patients (53.3%) had pyelonephritis, 10 (33.3%) had febrile UTI, 3 (10%) had prostatitis, and 1 (3.3%) had cystitis. Among patients with pyelonephritis, three (18.8%) presented with associated abscess formation (Figure 2). Additional patient-level clinical, microbiological, and treatment characteristics are provided in Supplementary Table S1.

### 2.2. Microbiological Findings (Figure 3)

At least one positive microbiological culture was obtained from 23 patients (76.7%). Eleven patients had positive urine cultures only (including one patient with a polymicrobial urine culture yielding two isolates), while eight had positive blood cultures only. Three patients had concordant positive blood and urine cultures, and one additional patient had both positive blood and urine cultures with different microorganisms isolated from the two specimens. Overall, 12 patients (40%) had UTI-associated bacteraemia.

**Figure 3:**
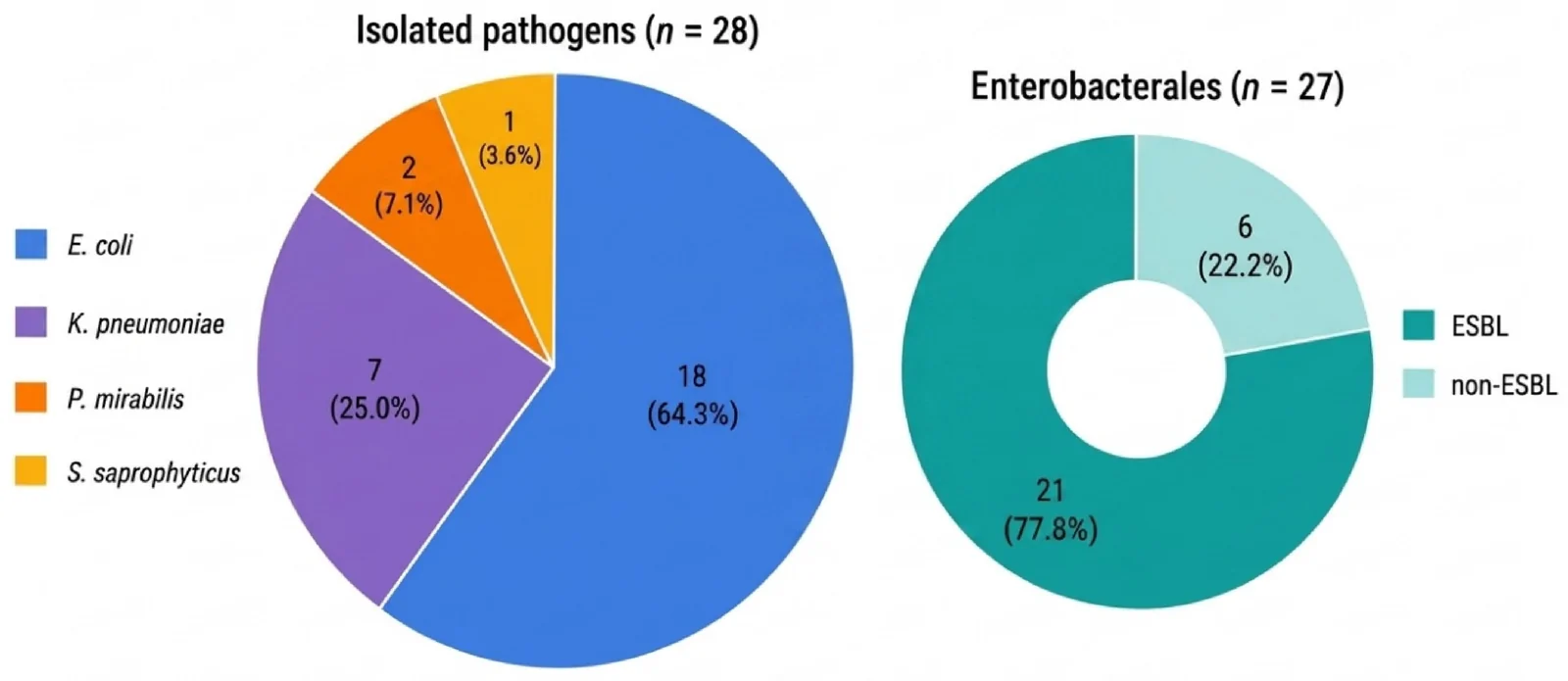
Distribution of isolated pathogens and ESBL phenotype among recovered isolates. Abbreviation: ESBL, extended-spectrum beta-lactamase.

A total of 28 bacterial isolates were recovered. *Escherichia coli* was the most frequently identified pathogen, accounting for 18 isolates (64.3%), followed by *Klebsiella pneumoniae* with 7 isolates (25%), *Proteus mirabilis* with 2 isolates (7.1%), and *Staphylococcus saprophyticus* with 1 isolate (3.6%). Overall, 21/27 Enterobacterales (77.8%) exhibited an ESBL-producing phenotype. At the patient level, infections involving at least one ESBL-producing isolate were identified in 17/30 patients (56.7%). Temocillin susceptibility testing was available for seven of the 28 isolates (25.0%), all of which were ESBL-producing Enterobacterales and were treated with temocillin as targeted therapy. MIC values were ≤4 mg/L in four isolates (two *E. coli* and two *K. pneumoniae*), 8 mg/L in two isolates (one *E. coli* and one *K. pneumoniae*), and 12 mg/L in one *E. coli* isolate, the latter determined by gradient diffusion.

### 2.3. Treatment and Management (Table 2)

Prior antimicrobial therapy had been administered in 22 patients (73.3%). Among the 23 patients with a microbiologically documented infection, the previous antimicrobial regimen was active against the subsequently identified isolate in 8 cases (34.8%).

**Table 2:** Characteristics of temocillin treatment and antimicrobial management.

Variable	Overall Cohort (*n* = 30)
Temocillin treatment regimen	
Treatment duration (days)	7.5 (7–10.5)
Daily dose (g/day)	6 (6–6)
6 g/day (*n*, %)	24 (80)
4 g/day (*n*, %)	4 (13.3)
1 g/day (*n*, %)	2 (6.7)
Continuous intravenous infusion (*n*, %)	23 (76.7)
OPAT (*n*, %)	5 (16.7)
Concomitant antimicrobial therapy	
Concomitant antimicrobial agent (*n*, %)	9 (30)
Fosfomycin disodium	2
Gentamicin	3
Amikacin	3
Doxycycline	1
Previous antimicrobial therapy	
Previous antimicrobial therapy (*n*, %)	22 (73.3)
Previous therapy active against the isolate (*n*/*N*, %)	8/23 (34.8)
Treatment modification	
Subsequent antimicrobial modification (*n*, %)	8 (26.7)
Switch to oral therapy	7
Escalation due to clinical failure	1

Data are presented as median (interquartile range [IQR]) for continuous variables and as number (percentage) for categorical variables, unless otherwise specified. Previous therapy active against the identified isolate was assessed only in patients with microbiologically documented infection (*n* = 23). Abbreviations: IQR, interquartile range; OPAT, outpatient parenteral antimicrobial therapy.

Regarding the clinical rationale for temocillin initiation, in 4 patients (13.3%) it was selected as a carbapenem-sparing strategy, either as de-escalation from a carbapenem or as an initial carbapenem-sparing option in patients with ESBL-producing Enterobacterales. In 10 patients (33.3%), temocillin was initiated following microbiological documentation of resistance to the previously prescribed antimicrobial regimen (nine of these infections were caused by ESBL-producing Enterobacterales). Temocillin was started empirically in 8 patients (26.7%). In the remaining 8 patients (26.7%), it was selected among other available non-carbapenem options based on the overall clinical and microbiological assessment, without a single predominant rationale identifiable retrospectively.

The median duration of temocillin treatment was 7.5 days (IQR 7–10.5), with a median daily dose of 6 g/day (IQR 6–6). The most prescribed regimen was 6 g/day, administered in 24 patients (80%); four patients received 4 g/day, whereas two patients with chronic renal impairment received 1 g/day (baseline serum creatinine 5.85 and 6.79 mg/dL, respectively). Temocillin was administered by continuous intravenous infusion in 23 patients (76.7%). Among these, 5 patients (16.7% of the overall cohort) continued treatment within an OPAT programme using Baxter INFUSOR™ LV elastomeric pumps (Baxter Healthcare Corporation, Round Lake, IL, USA; product code 2C1063KP). In these cases, temocillin was diluted in 240 mL of 0.9% sodium chloride solution and administered at a fixed infusion rate of 10 mL/h. At the time of transition to OPAT, all five patients were clinically stable, had pyelonephritis, and had no persistent manifestations of sepsis, UTI-associated bacteraemia, or other conditions requiring continued inpatient management. The median duration of outpatient treatment was 12 days (range 6–20), and clinical success was achieved in 4/5 patients (80%). No recurrence or death was documented within 30 days, and no adverse events considered attributable to temocillin occurred. One patient developed *C. difficile* infection within 60 days after treatment completion.

Temocillin was predominantly administered as monotherapy, with nine patients (30%) receiving at least one concomitant antimicrobial agent: intravenous fosfomycin in two cases, gentamicin in three, amikacin in three, and doxycycline in one.

Following temocillin treatment, eight patients underwent a modification of antimicrobial therapy. Seven were switched to an oral antimicrobial regimen after clinical improvement, whereas one patient required escalation to ertapenem because of clinical failure.

### 2.4. Clinical Outcomes (Table 3)

Overall, clinical success was achieved in 28 of 30 patients (93.3%; 95% CI 77.9–99.2%), including both patients with severe renal impairment who received temocillin at 1 g/day. When stratified by treatment strategy, clinical success was achieved in 19/21 patients receiving temocillin monotherapy (90.5%; 95% CI 69.6–98.8%) and in 9/9 patients receiving combination therapy (100%; 95% CI 66.4–100%). According to clinical presentation, clinical success was achieved in 15/16 patients with pyelonephritis (93.8%), 10/10 with febrile UTI (100%), 2/3 with prostatitis (66.7%), and 1/1 with cystitis (100%). Among patients with sepsis, clinical success was achieved in 14/15 patients (93.3%), including 8/9 receiving temocillin monotherapy (88.9%) and 6/6 receiving combination therapy (100%). Among patients with UTI-associated bacteraemia, clinical success was achieved in 11/12 patients (91.7%), including 9/10 receiving monotherapy (90%) and 2/2 receiving combination therapy (100%). Similarly, among patients with infections caused by ESBL-producing Enterobacterales, clinical success was achieved in 16/17 patients (94.1%), including 11/12 receiving monotherapy (91.7%) and 5/5 receiving combination therapy (100%).

**Table 3:** Clinical and safety outcomes of temocillin treatment.

Variable	Overall Cohort (*n* = 30)
Clinical outcomes	
Clinical success (*n*, %)	28 (93.3; 95% CI 77.9–99.2)
Overall clinical failure (*n*, %)	2 (6.7)
30-day recurrence (*n*, %)	0 (0)
30-day mortality (*n*, %)	0 (0)
Safety outcomes	
Acute kidney injury (*n*, %)	0 (0)
Electrolyte abnormalities (*n*, %)	0 (0)
Hepatotoxicity (*n*, %)	0 (0)
Neutropenia (*n*, %)	0 (0)
Thrombocytopenia (*n*, %)	0 (0)
Allergic reactions (*n*, %)	0 (0)
*Clostridioides difficile* infection within 60 days (*n*, %)	2 (6.7; 95% CI 0.8–22.1)

Clinical success in patients with UTI-associated bacteraemia required fulfillment of the predefined criteria for both urinary tract infection and bloodstream infection. Data are presented as numbers (percentage). *Clostridioides difficile* infections were assessed within 60 days after completion of temocillin therapy; recurrence and mortality were assessed within 30 days. Abbreviations: CI, confidence interval; UTI, urinary tract infection.

Two patients failed to meet the predefined criteria. One patient with culture-negative pyelonephritis had persistent lower back pain at the end of antibiotic therapy. A second patient with prostatitis and ESBL-producing *E. coli* bacteraemia, for which temocillin susceptibility testing was not available, experienced clinical failure requiring escalation to ertapenem. Of note, the patient with *Staphylococcus saprophyticus* isolated from urine had already shown a favourable clinical and laboratory response to temocillin before the urine culture result became available. Moreover, a few days after initiation of temocillin, doxycycline was also started following the molecular detection of *Chlamydia trachomatis* and *Mycoplasma hominis* in urine.

No recurrence of the index infection was documented within 30 days after completion of antibiotic therapy, and no deaths occurred within the 30-day follow-up period.

### 2.5. Safety and Adverse Events (Table 3)

No adverse events considered attributable to temocillin were documented, including among the two patients with severe renal impairment who received temocillin at 1 g/day. No episodes of acute kidney injury, clinically relevant electrolyte abnormalities, hepatotoxicity, neutropenia, thrombocytopenia, or allergic reactions were observed. Two patients developed *C. difficile* infection within 60 days after treatment completion (6.7%; 95% CI 0.8–22.1%). Of note, one of these patients had previously received piperacillin/tazobactam and intravenous fosfomycin, with fosfomycin subsequently continued in combination with temocillin.

## 3. Discussion

In this multicentre observational study, we evaluated the real-world use of temocillin in 30 patients with UTIs across three Italian hospitals. The study population included a substantial proportion of clinically complex cases, with 50% of patients presenting with sepsis and 40% with UTI-associated bacteraemia. Microbiological findings also reflected a high burden of antimicrobial resistance within the study population, as 56.7% of infections were caused by ESBL-producing pathogens, with *E. coli* representing the most frequently identified microorganism, followed by *K. pneumoniae*.

Despite the severity and complexity of the infections included, overall clinical success was achieved in 93.3% (95% CI 77.9–99.2%) of patients. These findings suggest that temocillin may be considered a potential treatment option in selected patients with UTIs, including some presenting with sepsis or UTI-associated bacteraemia. Although temocillin susceptibility testing was available for only a limited number of isolates, all tested strains remained within the susceptible-at-increased-exposure categories, including ESBL-producing isolates.

No adverse events considered directly attributable to temocillin were documented in this small cohort. Two patients developed *C. difficile* infection within 60 days after treatment completion. However, these events should be interpreted cautiously as a causal relationship with temocillin cannot be established.

Overall, our findings are in line with the available literature, which has generally reported favourable clinical outcomes with temocillin in UTIs, including complicated and bacteraemic cases. The overall clinical success rate observed in our cohort falls within the range reported in previous real-world and comparative studies. In the multicentre real-world study conducted in the Greater Paris area, Dinh et al. reported a Day-28 cure rate of 86.7% among 113 episodes caused by ESBL-producing Enterobacterales, with UTIs accounting for 85.8% of cases [11]. Similarly, Delory et al. evaluated 72 patients treated with temocillin for ESBL-producing Enterobacterales UTIs and reported a clinical cure rate of 94%, compared with 99% among matched patients receiving carbapenems, with no statistically significant difference between the two groups. Notably, bacteraemia was present in 36% of patients receiving temocillin, providing a population partially comparable to ours [12].

Further comparative data support these observations. In the propensity score-matched cohort reported by Iachkine et al., which included 180 patients with febrile UTIs caused by third-generation cephalosporin-resistant Enterobacterales, early clinical failure occurred in 9% of patients receiving temocillin compared with 14% receiving other intravenous β-lactams, without a significant difference between groups [13].

Evidence supporting temocillin in bloodstream infections has recently been strengthened by the ASTARTÉ randomized phase 3 trial. In this study, 328 patients with bloodstream infections caused by third-generation cephalosporin-resistant Enterobacterales were randomized to temocillin or carbapenems. Clinical success occurred in 74% of patients receiving temocillin and 73% receiving carbapenems, demonstrating non-inferiority of temocillin as targeted therapy. These results provide important high-level evidence supporting the carbapenem-sparing potential of temocillin in invasive infections [14].

In our study, most patients received 6 g/day, and continuous infusion was used in 76.7% of cases. Recent clinical PK/PD data in non-critically ill patients with complicated UTIs suggest that a 4 g/day regimen may provide insufficient target attainment in patients with preserved renal function, supporting the use of higher-exposure regimens in this setting [15]. Consistently, in the modelling study by Van Os et al., 6 g/day regimens and continuous infusion resulted in greater predicted bacterial killing than 4 g/day and intermittent administration, although regrowth of resistant subpopulations remained possible, particularly for strains with higher MICs [16].

The use of OPAT with elastomeric infusion pumps, which was adopted in 5 patients in our cohort (16.7%), represents a potential practical advantage of temocillin. Previous OPAT experiences have also reported favourable outcomes with temocillin in complicated UTIs. However, given the small number of patients treated through OPAT in the present study, these findings should be interpreted as a descriptive experience in selected clinically stable patients rather than as evidence supporting its general applicability [16,17].

Our microbiological findings are also consistent with the established activity of temocillin against β-lactamase-producing Enterobacterales. In our cohort, 77.8% of Enterobacterales were ESBL producers, and all seven isolates for which temocillin susceptibility testing was available were classified as susceptible at increased exposure. Although the small number of tested isolates precludes broader conclusions, these findings are consistent with studies reporting preserved activity against ESBL-producing organisms. No AmpC-producing Enterobacterales were identified in our cohort. However, previous studies have also reported substantial in vitro and clinical activity of temocillin against AmpC-producing isolates. In particular, Brousse et al. found that 82.3% of 5166 AmpC-producing Enterobacterales isolates were susceptible to temocillin, increasing to 85.8% among urinary isolates [18].

Finally, no adverse events considered directly attributable to temocillin were documented in this small cohort. During follow-up, two patients developed *C. difficile* infection within 60 days after treatment completion (6.7%), a rate comparable with that reported in other clinical studies of temocillin, including the ASTARTÉ trial and the cohort by Delory et al., both of which reported rates of approximately 6% [12,14].

This study has several strengths. To our knowledge, it provides the first multicentre real-world description of temocillin use for UTIs in Italy and includes patients treated across three different hospitals, thereby reflecting heterogeneous routine clinical practice. The cohort included clinically relevant cases characterized by sepsis, bloodstream infection, and ESBL-producing pathogens. Furthermore, the collection of treatment-related variables allowed the evaluation of different administration strategies, including continuous infusion and OPAT through elastomeric pumps, together with predefined clinical and safety outcomes.

On the other hand, several limitations should also be acknowledged. First, the small sample size limits the precision of the estimates and the generalizability of our findings, while also precluding meaningful subgroup analyses. Second, the retrospective observational design and the absence of a comparator group do not allow direct assessment of the effectiveness of temocillin relative to alternative treatments. Third, the inclusion of only patients who received temocillin for at least 5 days may have introduced selection bias, as patients experiencing very early treatment failure, treatment discontinuation, or death could have been excluded. This may have preferentially selected patients able to remain on treatment and potentially led to an overestimation of favourable clinical outcomes and tolerability. Fourth, temocillin susceptibility testing was available for only a limited proportion of the isolated pathogens. In addition, heterogeneity in local microbiological and clinical practices across participating centres may have influenced diagnostic and therapeutic management. Finally, the relatively short follow-up period limits the assessment of late recurrence and longer-term safety outcomes. Overall, these findings should be considered descriptive and should not be interpreted as definitive evidence of clinical efficacy.

## 4. Materials and Methods

### 4.1. Study Design and Population

This retrospective multicentre observational study was conducted at three Italian hospitals: the University Hospital (ASUGI) of Trieste, Santa Maria degli Angeli Hospital (Pordenone), and Santa Chiara Hospital (Trento). Adult patients treated with temocillin between November 2024 and May 2026 were identified through Infectious Diseases service records, and eligibility was subsequently verified through review of the medical records. Eligible patients were aged ≥ 18 years, had a diagnosis of UTI with or without concomitant bloodstream infection, and received temocillin therapy for at least 5 days. Pregnant women were excluded. Sepsis was identified based on the contemporaneous clinical diagnosis documented by the treating physicians, supported by evidence of acute organ dysfunction considered attributable to the UTI. Because the complete set of variables required for SOFA calculation was not consistently available in the retrospective medical records, a formal retrospective SOFA-based reclassification was not performed.

All patients provided informed consent for the data collection. All data were pseudonymized via a web-based central, password-protected clinical database management system.

### 4.2. Data Collection and Statistical Analysis

Demographic characteristics, medical history, microbiological findings, treatment characteristics, and clinical outcomes were collected from electronic medical records and entered into a dedicated database. Data were analysed using descriptive statistics. Continuous variables are presented as median and interquartile range (IQR), whereas categorical variables are reported as absolute numbers and percentages. For proportions of the main clinical outcomes, 95% confidence intervals were calculated using the exact binomial (Clopper–Pearson) method.

### 4.3. Microbiological Methods

Urine and blood cultures were processed according to routine laboratory procedures at each participating centre. Pathogen identification was performed by matrix-assisted laser desorption/ionization time-of-flight mass spectrometry (MALDI-TOF MS, VITEK MS; bioMérieux, Marcy-l’Étoile, France).

Antimicrobial susceptibility testing and resistance phenotype characterization were performed in accordance with local laboratory practices. In particular, temocillin susceptibility testing was not available at one participating centre and was progressively implemented at the other two centres during the study period. When performed, testing was carried out either by gradient diffusion (MTS, Liofilchem S.r.l., Roseto degli Abruzzi, Italy) or using the VITEK 2 automated system (AST-XN26 card, bioMérieux, Marcy-l’Étoile, France), according to local laboratory practice. Antimicrobial susceptibility results were interpreted according to the current European Committee on Antimicrobial Susceptibility Testing (EUCAST) clinical breakpoints. For Enterobacterales, EUCAST defines temocillin MIC values of 0.001–16 mg/L as susceptible at increased exposure (I), whereas isolates with MIC values > 16 mg/L are categorized as resistant (R). These breakpoints apply to a high-exposure dosing regimen of 2 g intravenously every 8 h (6 g/day) and are intended for complicated and more severe UTIs, including urosepsis, but not severe sepsis or septic shock [19,20].

### 4.4. Study Outcomes

The primary objective of the study was to describe the clinical and microbiological characteristics of patients treated with temocillin for UTIs in routine clinical practice. Secondary objectives were to evaluate the clinical outcomes and tolerability associated with temocillin treatment. Clinical outcomes were retrospectively adjudicated by the study investigators according to predefined criteria, based on signs and symptoms documented in electronic medical records.

Clinical success in patients with UTI was defined as complete resolution of urinary symptoms, fever, and flank or lower back pain by the end of antibiotic therapy. In patients with bloodstream infection, clinical success was defined as completion of an appropriate course of antibiotic without the need to restart antibacterial therapy within 48 h after treatment discontinuation because of recurrence of the bloodstream infection. For patients presenting with both UTI and concomitant bloodstream infection, clinical success required fulfilment of both definitions. Follow-up blood cultures were not routinely obtained, and microbiological clearance was therefore not included in the definition of clinical success. For patients who underwent oral step-down therapy after clinical improvement, clinical success was assessed at the end of the overall antibiotic treatment course.

Recurrence of the index infection and all-cause mortality were assessed within 30 days after completion of antibiotic therapy through review of medical records from the participating centres. Safety outcomes included adverse events considered by the treating physicians to be attributable to temocillin, including acute kidney injury (increase in serum creatinine ≥ 0.3 mg/dL or 1.5–1.9 times baseline values), clinically relevant electrolyte abnormalities (serum sodium < 135 or >145 mEq/L and/or serum potassium < 3.5 or >5.0 mEq/L), hepatotoxicity (AST and/or ALT > 3 times the upper limit of normal), neutropenia (absolute neutrophil count < 1500 cells/mm^3^), thrombocytopenia (platelet count < 150,000 cells/mm^3^), and allergic reactions occurring during treatment. *Clostridioides difficile* infection occurring within 60 days after completion of temocillin therapy was collected separately as a predefined post-treatment safety outcome, irrespective of its assessed causal relationship with temocillin. Clinical suspicion and diagnosis of *C. difficile* infection were established according to the 2017 IDSA/SHEA Clinical Practice Guidelines [21].

### 4.5. Ethical Approval

The study protocol was reviewed and approved by the Ethics Committee of the University of Trieste (n°V8_240925). The planning, conduct and reporting of this study were in line with the Declaration of Helsinki.

## 5. Conclusions

This multicentre real-world study provides initial Italian data on the use of temocillin for UTIs. In this small cohort, temocillin was associated with favourable clinical outcomes, with no adverse events considered attributable to treatment, including among selected patients with sepsis, UTI-associated bacteraemia, and infections caused by ESBL-producing Enterobacterales. However, no AmpC-producing Enterobacterales were identified in our cohort, and therefore no conclusions can be drawn regarding this subgroup.

Moreover, continuous infusion and OPAT through elastomeric pumps were used in selected clinically stable patients, allowing completion of parenteral treatment outside the hospital setting in appropriately selected cases.

Although these findings are limited by the small sample size and observational design, they support further evaluation of temocillin as a potential carbapenem-sparing option for complicated UTIs.

## Data Availability

The original contributions presented in the study are included in the article/Supplementary Materials; further inquiries can be directed to the corresponding author.

## Supplementary Materials

The following supporting information can be downloaded at: https://www.mdpi.com/article/10.3390/antibiotics15101034/s1, Table S1: Supplementary clinical, microbiological, and treatment data of the study population.

## Abbreviations

The following abbreviations are used in this manuscript:

CCI	Charlson Comorbidity Index
CRP	C-reactive protein
ESBL	Extended-spectrum β-lactamase
EUCAST	European Committee on Antimicrobial Susceptibility Testing
MALDI-TOF MS	Matrix-assisted laser desorption/ionization time-of-flight mass spectrometry
MIC	Minimum inhibitory concentration
OPAT	Outpatient parenteral antimicrobial therapy
PCT	Procalcitonin
UTI	Urinary tract infection
WBC	White blood cell count
